# Dynamic contrast enhanced (DCE) MRI estimation of vascular parameters using knowledge-based adaptive models

**DOI:** 10.1038/s41598-023-36483-9

**Published:** 2023-06-14

**Authors:** Hassan Bagher-Ebadian, Stephen L. Brown, Mohammad M. Ghassemi, Tavarekere N. Nagaraja, Olivia Grahm Valadie, Prabhu C. Acharya, Glauber Cabral, George Divine, Robert A. Knight, Ian Y. Lee, Jun H. Xu, Benjamin Movsas, Indrin J. Chetty, James R. Ewing

**Affiliations:** 1 Department of Radiation Oncology, Henry Ford Health, Detroit, MI 48202 USA; 2 Department of Radiology, Michigan State University, East Lansing, MI 48824 USA; 3 Department of Osteopathic Medicine, Michigan State University, East Lansing, MI 48824 USA; 4 Department of Physics, Oakland University, Rochester, MI 48309 USA; 5 Department of Computer Science and Engineering, Michigan State University, East Lansing, MI 48824 USA; 6 Department of Neurosurgery, Henry Ford Health, Detroit, MI 48202 USA; 7 Department of Radiation Oncology, Wayne State University, Detroit, MI 48202 USA; 8 Department of Neurology, Henry Ford Health, Detroit, MI 48202 USA; 9 Department of Public Health Sciences, Henry Ford Health, Detroit, MI 48202 USA; 10 Department of Epidemiology and Biostatistics, Michigan State University, E. Lansing, MI 48824 USA; 11 Department of Neurology, Wayne State University, Detroit, MI 48202 USA

**Keywords:** Cancer, Cancer imaging, Computational science, Mathematics and computing, Statistics

## Abstract

We introduce and validate four adaptive models (AMs) to perform a physiologically based Nested-Model-Selection (NMS) estimation of such microvascular parameters as forward volumetric transfer constant, K^trans^, plasma volume fraction, v_p_, and extravascular, extracellular space, v_e_, directly from Dynamic Contrast-Enhanced (DCE) MRI raw information without the need for an Arterial-Input Function (AIF). In sixty-six immune-compromised-RNU rats implanted with human U-251 cancer cells, DCE-MRI studies estimated pharmacokinetic (PK) parameters using a group-averaged radiological AIF and an extended Patlak-based NMS paradigm. One-hundred-ninety features extracted from raw DCE-MRI information were used to construct and validate (nested-cross-validation, NCV) four AMs for estimation of model-based regions and their three PK parameters. An NMS-based a priori knowledge was used to fine-tune the AMs to improve their performance. Compared to the conventional analysis, AMs produced stable maps of vascular parameters and nested-model regions less impacted by AIF-dispersion. The performance (Correlation coefficient and Adjusted R-squared for NCV test cohorts) of the AMs were: 0.914/0.834, 0.825/0.720, 0.938/0.880, and 0.890/0.792 for predictions of nested model regions, v_p_, K^trans^, and v_e_, respectively. This study demonstrates an application of AMs that quickens and improves DCE-MRI based quantification of microvasculature properties of tumors and normal tissues relative to conventional approaches.

## Introduction

Dynamic Contrast Enhanced Magnetic Resonance Imaging (DCE-MRI) allows for the quantification of microvascular parameters such as the forward volumetric transfer constant (K^trans^), plasma volume (v_p_), and extra-vascular extracellular space (v_e_). These vascular parameters are being evaluated for their value in cancer treatment planning and prediction of tumor aggressiveness^1^. In animal models, DCE-MRI Pharmacokinetic (PK) analysis shows promise in describing tumor physiology^2,3^, and of physiological reaction to treatment^4–10^.

Box’s heuristic that “All models are wrong, some are useful^11^” rules DCE-MRI studies in brain. A thorough consideration of systematic sources of errors in an experiment conducted in the brain of small animals at 7 Tesla examined a number of sources, including T2* dephasing, intervoxel contrast exchange, water exchange effects, arterial input function (AIF) dispersion, and over- or under-fitting of concentration–time data^12^. It can be concluded that T2* dephasing can be compensated for by using Dual Gradient-Echo DCE-MRI, and water exchange effects can be minimized by choice of tip-angle and repetition time^13^, yielding reasonable confidence that the change in the longitudinal relaxation rate ΔR_1_(t) (R_1_ = 1/T_1_) is directly proportional to the concentration of contrast agent in the tissue. Of the other *known* sources of systematic error, a data-driven approach to model selection will at least avoid over- or under-fitting^12,14^.

That said, it is clear from an examination of the data that there are elements in modeling DCE-MRI that are not accounted for (c.f. Fig. 8 of Ewing and Bagher-Ebadian) and cannot be accounted for if over-fitting is to be avoided. These ‘tapering effects’ are typical in modeling of all biological systems^15^. The two most evident sources of error are errors in estimating the true arterial contrast agent concentration, and dispersion due to flow of the arterial input function (AIF) as it progresses from the large arteries to the capillary bed, where exchange with the extravascular space might take place. Estimating the true AIF concentration presents significant difficulties, with inflow, outflow, and partial-volume effects undermining efforts at measurement. This, and dispersion in the arterial tree^16,17^ substantially undermine the assumption that either the amplitude or the shape of the tissue input function can be determined from a time trace of arterial contrast. One approach to estimating input amplitude is to employ a group-averaged time trace of arterial CA concentration normalized to a known vascular volume in normal brain tissue^12,18^, thus deliberately introducing a bias that is presumably smaller than the bias of measuring contrast change directly from a large artery and inferring dynamic contrast agent concentration. This does not address problems with AIF dispersion.

Parameters of microvasculature and perfusion in normal tissue and tumor in PK models are usually estimated by forming a cost function based on the parameters and sampled data and using optimization methods to minimize the cost function^19–23^. These optimization/fitting methods include nonlinear least-squares (NLLSQ) fitting^20^, linear least-squares (LLSQ)^21,22^, and an efficient derivative-based LLSQ method with a low-pass filter in the time domain^23^. The literature reports^19–24^ that these methods are computationally intensive and sensitive to sampling interval, total acquisition time, and noise. For the methods developed so far, an obvious degradation of performance with increasing noise level and decreasing temporal resolution is observed.

In recent years, adaptive methods have been investigated by multiple groups in an effort to generate more accurate and stable estimates of PK vascular parameters by extracting time-dependent features from DCE-MRI^25^. Bliesener et al.^26^ estimated a voxel-wise map of K^trans^ from DCE-MRI data using deep learning (DL) method with one-dimensional convolutional neural network (CNN) architecture. They used contrast agent concentration maps and arterial input function information as input to the network. Cagdas et al.^27,28^ estimated the PK parameters using dilated convolution and fully connected layers with a channel-wise temporal dimension model. Kettelkamp et al.^29^ introduced a modified version of the network proposed by Cadgas et al. by incorporating the AIF information as input to the network. However, as the proposed networks recruit fully convolutional layers and treat the temporal information as a separate dimension or channel to their inputs, they are susceptible to the temporal information change of the data and its corresponding AIF information which require the adaptive models to retrain these changes for accurate prediction. Kettelkamp et al.^29^ also estimated the PK parameters from fully sampled DCE-MRI data with explicit knowledge of AIF. Rastogi et al.^30^ have recently proposed an adaptive model with different architecture to address the issues of the information retention (spatiotemporal dimension) requirement as well as the need for the AIF information for PK parameter estimation. They proposed a 2.5D Unet-based architecture for direct estimation of the PK parameters from under-sampled *k-t* space.

**Figure 1:**
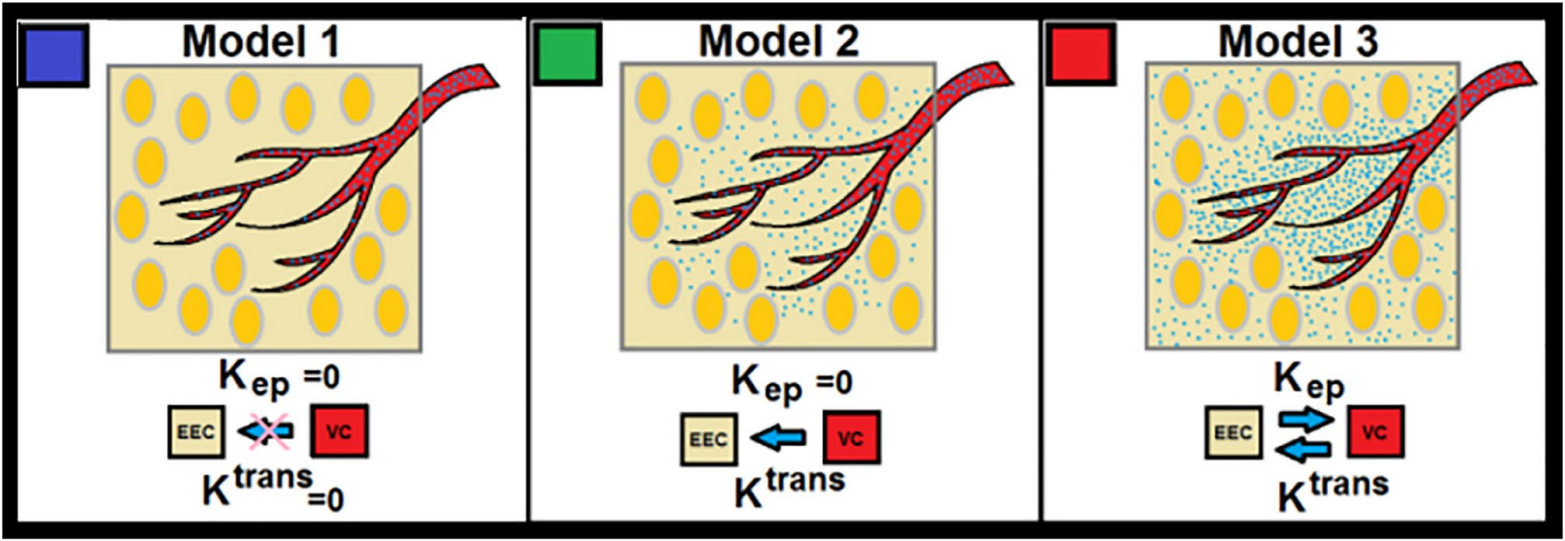
This figure illustrates three physiologically nested models and their estimable vascular parameters corresponding to three different observation equations (Eqs. 10–12).

Despite considerable efforts by these groups, less attention has been paid to the important matter of the tracer kinetic *‘*model selection^1,12,14,31–34^ in processing that physiologically associates estimable vascular parameters with relevant information available in the DCE-MRI signal. Our group has taken an important step toward the improvement of the image-based estimation of vascular parameters by introducing a nested model selection (NMS) technique^12,14^ utilizing the Patlak graphical method and its extension^12,14^ in pharmacokinetic (PK) compartmental analyses of DCE-MRI data from rat and human brains. The NMS generates maps of brain regions and labels them with the number of estimable parameters (see Fig. 1). However, identification of model choice regions requires a series of computationally intensive processes, and the estimates of the vascular parameters can be strongly affected by an investigator’s choices in the different stages of experimental design and the analysis of data.

This study introduces a series of knowledge-based adaptive models in the form of an artificial neural networks (ANNs) motivated by the NMS concept to predict model choice regions and estimate their respective PK parameters directly from the raw DCE-MR information without the need for AIF selection, and with less sensitivity to AIF dispersion. This study is the first to develop AM predictors of physiological parameters of brain tumors in an animal model that not only addresses the complexity of processing, but also recruits standard physiologically nested model selection techniques^12,14^ to improve reproducibility and decrease bias in conventional compartmental modeling in PK analysis of DCE MRI data.

## Methods

### Ethical approval

All experimental and imaging parts of the study were conducted at our institution based on an approved protocol by the Institutional Animal Care and Use Committee (IACUC) of Henry Ford Hospital. The approval number was 1509. The animal study of this work was performed and reported in compliance with the ARRIVE guidelines. Also, all methods were performed in accordance with the relevant guidelines and regulations. All animals were put in anesthesia before and during MRI experiment using Isoflurane which is a volatile anesthetic approved by the Federal Drug Administration (FDA). They were also sacrificed with Ketamine using an approved IACUC (#1509) protocol.

### Animal population and DCE-MR imaging

Following previously published methods, and in an institutionally approved protocol, 66 immune-compromised-RNU/RNU rats were stereotactically implanted with 5 × 10^5^ U-251 cancer cells in 10 µl of phosphate buffered saline (PBS) that formed a 3–4 mm diameter orthotopic glioma about 2 weeks later^35^. Sixty-six DCE-MRI studies (multi-slice, multi-echo gradient-echo sequence, with three 2.0 mm slices, no gap, matrix: 128 × 64, FOV:32 × 32 mm^2^, T_R_/(T_E1_ − T_E2_) = 24 ms/(2 ms − 4 ms), flip-angle = 18°, SW = 150 kHz) were performed using a 7 T Varian (Agilent, 20 cm bore system with Bruker console) scanner. Four-hundred slice packages at ~ 1.55 s intervals (experiment duration: 10 min) were acquired. A bolus of magnetic-resonance contrast-agent (MR CA-Magnevist) was injected (tail-vein) by hand push at acquisition 15. This study was conducted with an approved IACUC # 1509.

### Nested model selection and observation equations for pharmacokinetic analysis

The Standard Model (SM) is a starting point for evaluating cerebral physiology^12,14,36^ following measurement of contrast tracers using DCE-MRI experiment. Modeling of leakage in the vasculature system in integral form was first generated by Patlak^37,38^ and further modified by Tofts et al.^39,40^. After intravenous administration of CA, the CA concentration in tissue is given by:

1 $${\text{C}}_{t}\left(t\right)={K}^{trans}\int \limits_{0}^{t}{{\text{e}}^{-{k}_{ep}\left(\text{t}-\uplambda \right)}\text{C}}_{p}\left(\lambda \right)d\lambda +{v}_{p}{C}_{p}\left(t\right),$$

where C_p_ and C_t_ are the plasma and tissue concentrations of the CA, K^trans^ is the volumetric forward transfer rate of the indicator into the interstitial space. The k_ep_ in Eq. (1) refers to the transfer rate from the interstitial compartment to the vascular compartment, t and λ denote the experiment time, and v_p_ is the fractional volume of the plasma distribution space. As illustrated in Fig. 1, we have shown^12,14^ that three physiologically nested models can be derived from the standard model to describe possible physiological conditions of underlying tissue pathology^12,14^. We have generated a series of stable processing pipelines accordingly, for producing vascular parametric maps based on the NMS^12,14^. We assume that CA concentration, [Gd], is proportional to the change in the longitudinal relaxation rate (R_1_) after CA administration: [Gd] = CA(t) ~ ΔR_1_(t), where R_1_ = 1/T_1_ and T_1_ is the longitudinal relaxation time. This assumption is supported in most DCE-MRI studies because they are conducted under the conditions of rapid repetition time and Ernst tip-angle adjusted for a mean decrease in R_1_. Under these conditions, studies in multicompartmental systems demonstrate little effect of water exchange mechanisms^41,42^.

### Calculation of CA concentration (∆R_1_) from the dual gradient echo (DGE) pulse sequence

DGE imaging allows for the computation of *T*_*1*_ and *T*_2_* as approximately pure and independent components. This is essential for the estimation of CA concentration based on *ΔR*_*1*_ and *ΔR*_2_*. Below, we describe the method we have developed for calculating the *ΔR*_*1*_ signal (~ CA concentration) from the DGE signals. The equations describe the measured T_1_-weighted intensities of the first and second echo signals and their relationship to the longitudinal and transverse relaxation times (*T*_*1*_*, T*_2_^*^), repetition time (*T*_*R*_), echo time (*T*_*E*_), flip angle (*θ*), and equilibrium longitudinal magnetization (*M*_*0*_). (T_2_* includes the effects of static dephasing). *F* and S in these equations denote the signal from the 1st and 2nd echoes, respectively:

2 $$F=\frac{{M}_{0}\text{sin}\theta \left(1-{e}^{\frac{-TR}{T1}}\right){e}^{\frac{-TE1}{{T}_{2}^{*}}}}{1-{\text{cos}\theta e}^{\frac{-TR}{T1}}},$$

3 $$S=\frac{{M}_{0}\text{sin}\theta \left(1-{e}^{\frac{-TR}{T1}}\right){e}^{\frac{-TE2}{{T}_{2}^{*}}}}{1-{\text{cos}\theta e}^{\frac{-TR}{T1}}},$$

4 $$\mu =\frac{TE2}{TE1}.$$

For each voxel, there are two-time courses for the signal: the pre-injection time, and the post injection time. To set a baseline for the signal intensity of each voxel prior to the injection of the CA, the voxel intensities for the ${S}_{t}$ and ${F}_{t}$ signals are averaged over a few time points prior to injection:

5 $${F}^{pre}=\frac{1}{(n-m+1)}\sum_{t=m}^{n}{F}_{t}, { F}^{sat}=\frac{1}{(p-o+1)}\sum_{t=o}^{p}{F}_{t},$$

6 $${S}^{pre}=\frac{1}{(n-m+1)}\sum_{t=m}^{n}{S}_{t}, { S}^{sat}=\frac{1}{(p-o+1)}\sum_{t=o}^{p}{S}_{t}.$$

In Eqs. (5) and (6), *t* denotes the experiment time point and ${F}^{pre}$, and ${S}^{pre}$ are the mean of the signal intensities associated with first and second echoes between time points *m* and *n,* where *m* and *n* are smaller than the injection time. In these equations, ${F}^{Sat}$, and ${S}^{Sat}$ are the mean of the signal intensities associated with first and second echoes (saturated part of the signals) between time points *o* and *p*, where *p* is the last time point of the signals (post injection), and *p* minus *o* is equal to *n* minus *m* (*p − o* = *n − m*).

Also, we define ${E}^{pre}$ and ${E}^{post}$ as:

7 $${{E}^{pre}=e}^{\frac{-TR}{T1(pre)}}, {{E}^{post}=e}^{\frac{-TR}{T1(post)}},$$

which are *T*_*1*_ associated parameters, estimated from the *T*_*1*_ mapping pulse sequences that are run before and after the DCE-MRI study. Using the above definitions, the following equations for the preprocessing of the DGE signals for DCE imaging are:

8 $${\theta }_{\mathit{Est}}= {\text{cos}}^{-1}\left\{\frac{1-\left[\frac{{\left( \sum_{t=m}^{n}{S}_{t}^{pre} \right)}^{ \frac{1}{\mu -1}}}{{\left( \sum_{t=m}^{n}{F}_{t}^{pre} \right)}^{ \frac{\mu }{\mu -1}}}\right]\left[\frac{{\left( \sum_{t=m}^{n}{F}_{t}^{sat} \right)}^{ \frac{\mu }{\mu -1}}}{{\left( \sum_{t=m}^{n}{S}_{t}^{sat} \right)}^{ \frac{1}{\mu -1}}}\right]}{{E}^{post}-{E}^{pre}\left[\frac{{\left( \sum_{t=m}^{n}{S}_{t}^{pre} \right)}^{ \frac{1}{\mu -1}}}{{\left( \sum_{t=m}^{n}{F}_{t}^{pre} \right)}^{ \frac{\mu }{\mu -1}}}\right]\left[\frac{{\left( \sum_{t=m}^{n}{F}_{t}^{sat} \right)}^{ \frac{\mu }{\mu -1}}}{{\left( \sum_{t=m}^{n}{S}_{t}^{sat} \right)}^{ \frac{1}{\mu -1}}}\right] }\right\} ,$$

9 $${\Delta R}_{1}\left(t\right)= \frac{-1 }{TR}\text{ln}\left\{\frac{1}{\text{cos}{\theta }_{Est}{E}^{pre}}+\left(n-m+1\right)\left[1- \frac{1}{\text{cos}{\theta }_{Est}{E}^{pre}}\right]\left\{\frac{{\left[ \sum_{t=m}^{n}{S}_{t}^{pre} \right]}^{ \frac{1}{\mu -1}}}{{\left[ \sum_{t=m}^{n}{F}_{t}^{pre} \right]}^{ \frac{\mu }{\mu -1}}}\right\}\left\{\frac{{\left[{ F}_{t} \right]}^{ \frac{\mu }{\mu -1}}}{{\left[{ S}_{t} \right]}^{ \frac{1}{\mu -1}}}\right\}\right\}.$$

In these equations, for each voxel, the pure component ${\Delta R}_{1}\left(t\right)$, along with the local tip angle are to be estimated. An interesting observation about this equation is that the estimated ${\Delta R}_{1}\left(t\right)$ is not dependent on the value of ${E}^{post}$. However, a similar equation can be written based on ${E}^{post}$, and a balanced fitting procedure can be implemented if both ${E}^{pre}$ and ${E}^{post}$ are measured. In this study, the voxel-wise profiles of the CA concentration map (${\Delta R}_{1}\left(t\right)$) were directly estimated from these equations during the DCE-MRI experiment and the pre and post *T*_*1*_ associated parameters (${E}^{pre}$, and ${E}^{post}$) estimated by pre- and post-DCE Look-Locker sequences.

In this study, given Eq. (9), the time trace of change in the longitudinal relaxation time (ΔR_1_) in all the voxels of the animal’s brain, corresponding to the time trace of contrast agent concentration, for 66 DCE-MRI studies were calculated^14^. Post-processing and pharmacokinetic compartmental analyses of DCE-MRI data were carried out following published methods^12,14^, initially using a nested model selection (NMS)^12,14^ paradigm based on Patlak and extended Patlak graphical methods^37,38,43^. As shown in Fig. 1, the NMS technique^12,14^ was used to generate maps of brain regions labeled with the number of parameters used to describe the data^12^: (a) Model 1 region: normal vasculature with no leakage, the only parameter estimated is plasma volume, v_p_; (b) Model 2 region: tumor tissues with CA leakage without measurable back-flux to the vasculature, in which case, v_p_ and, K^trans^ can be estimated; or (c) Model 3 region: tumor vessels with CA leakage and measurable back-flux and, thus, v_p_, K^trans^, and k_ep_, or extracellular extra-vascular volume, v_e_ (ratio of K^trans^ and K_ep_) can be estimated. Three observation equations (see Eqs. 10–12) corresponding to three physiologically nested models were constructed from Eq. (1) as follows:

10 $${\text{C}}_{tissue}\left(\text{t}\right)=\frac{1}{1-{H}_{ct}}\left[{{v}_{p}\text{C}}_{AIF}\left(\text{t}\right)\right],$$

11 $${\text{C}}_{tissue}(\text{t})=\frac{1}{1-{H}_{ct}}[{{v}_{p}\text{C}}_{AIF}\left(\text{t}\right)+{K}^{trans}{\int }_{0}^{t}{\text{C}}_{AIF}\left(\lambda \right)d\lambda ],$$

12 $${\text{C}}_{tissue}\left(\text{t}\right)=\frac{1}{1-{H}_{ct}}\left[{{v}_{p}\text{C}}_{AIF}\left(\text{t}\right)+{K}^{trans}{\int }_{0}^{t}{\text{C}}_{AIF}\left(\lambda \right){e}^{-{K}_{ep}\left(t-\lambda \right)}d\lambda \right],$$

where *C*_*AIF*_* (t) and C*_*tissue*_*(t)* refer to the contrast agent (CA) concentration measured from whole blood (or arterial input function, AIF) and tissue of interest in the brain, respectively. The *Hct* in these equations refers to the hematocrit of the animal’s blood.

For each observation equation, the voxel by voxel time trace of ${\Delta R}_{1}\left(t\right)$ in rat brain was used to estimate its PK parameters as well as constructing an AIF for the brain^43^, based on a group averaged radiological trace of CA concentration in arterial blood^43^. At image 15 (corresponds to ~ 23 s after the start of the DCE experiment) of the dual GE sequence, a bolus injection of the CA (Magnevist; Bayer HealthCare LLC, Wayne, NJ, 0.25 mmol/kg at undiluted concentration, no flush) was performed. The group averaged radiological trace was normalized to the time trace of CA concentration in the animal’s normal caudate putamen^43^, with the assumption that plasma volume fraction in caudate putamen is 1%. Then, the normalized radiological AIF was used as *C*_*p*_ (CA concentration measured from plasma) or $\frac{{C}_{AIF}}{1-Hct}$ in Eqs. (10) to (12). A Simplex optimization/algorithm^44^ was used to estimate PK parameters for each model. Our experience^3,12,14,16,17,31,42,45–51^ with the Simplex optimization is that it is time-intensive but reliable in converging to a best-fit value.

### DCE-MRI feature engineering, adaptive model training and validation

As each voxel of the animal’s brain belonged to a model choice, to maintain the signal ratio of the two echoes, the last 10 timepoints of the raw signal intensity of the 1st echo, *F*_*e*_*(x, y, z, t),* were averaged (*t* = 391 to 400) and used as the normalization factor (*N*_*fac*_, see Eq. 13) for its 1st and 2nd echo profiles.

To reduce the dimensionality of the features and to allow the signal to settle down to a steady-state prior to the injection of CA, for each voxel, the first 20 timepoints of the normalized profiles (1st and 2nd echoes) were completely eliminated. Then, the signals were interleaved or down-sampled (Eqs. 14, 15) by a factor of 4 (~ 6 s), and used as the normalized feature set, *µ (x, y, z, k* = 1 to 190) for model development and validation parts of the study:

13 $${N}_{fac}\left(x,y,z\right)=\left(\frac{1}{{N}_{Tail}}\right)\sum_{t={n}_{t}}^{{N}_{0}}Fe\left(x,y,z,t\right), {N}_{Tail}={N}_{0}-{n}_{t}+1, {N}_{0}=400, and \,{n}_{t}=391,$$

14 $$for 1\le k\le 95, \mu \left(x,y,z, k\right)=\left\{\frac{Fe\left(x,y,z,{ L}_{1}+\left[4\times \left(k-1\right)+1\right]\right)}{{N}_{fac}\left(x,y,z\right)}\right\}, {L}_{1}=20,$$

15 $$for 96\le k\le 190, \mu \left(x,y,z, k\right)=\left\{\frac{Se\left(x,y,z,{ L}_{1}+\left[4\times \left(k-1\right)+1\right]-95\right)}{{N}_{fac}\left(x,y,z\right)}\right\}, {L}_{1}=20,$$

where *Fe(x, y, z, t)* and *Se(x, y, z, t)* refer to 1st and 2nd echo DCE-MRI profiles measured at timepoint *t* from a voxel of rat brain located at (x, y, z), and N_fac_(x,y,z) in Eq. (13) refers to the spatial normalization factor. Equations (14) and (15) define the spatiotemporal feature set (*µ (x, y, z, k), k* = 1 to 190) of each voxel by merging the two normalized down-sampled DCE-MRI components (1st and 2nd) together. A series of normalized dynamic feature sets (*µ)* were extracted from the 1st and 2nd echoes of the DCE-MRI signal of rat brains for all 66 studies and were used to train and validate four different adaptive models (AMs). The first AM was trained and validated (in their performance) as a regressor and classifier both to execute a voxel-wise nested model selection classification; the other three AMs were trained and validated to estimate the three PK parameters (v_p_, K^trans^, and v_e_). Using the set of features ($\mu (x,y,z,k)$ above) extracted from raw DCE-MRI information of 66 studies, four shallow artificial neural networks (ANNs, with feed-forward perceptron architectures, Levenberg–Marquardt optimization^52^, targets: NMS regions and PK parameters) were separately constructed, trained, and validated to perform NMS as well as estimate the three NMS-based PK parameters, respectively. The ANNs in this study are considered shallow ANNs since they have only one hidden layer. The NMS and PK analysis results of the standard Patlak analysis were used as the source of truth for training the adaptive models. The three ANNs for estimating the three PK parameters were constructed based on the NMS results generated by the first ANN which was responsible for the nested model selection.

To suppress AIF-dispersion and gradient errors, a knowledge-based optimization was performed on the ANNs’ response distributions to find the best class separation thresholds with a 5% miss-classification tolerance between each pair of the nested classes (Model 1 versus Model 2, and Model 2 versus Model 3).

Finally, all the adaptive models (the four ANNs) were validated using tenfold nested cross validation (NCV) technique^53^.

### Optimization of ANNs, estimation of ANNs’ performance and generalization error

**Figure 2:**
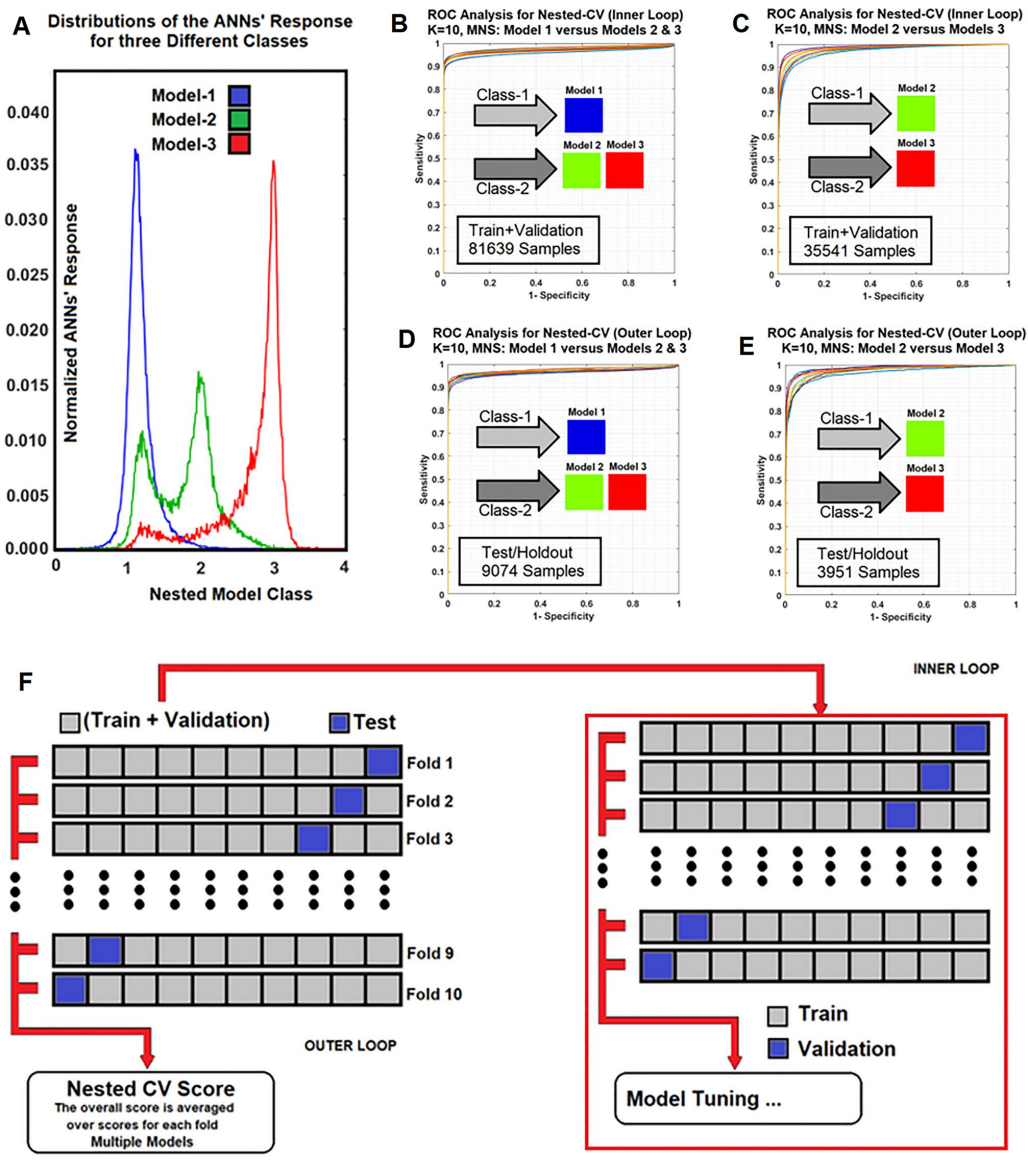
(**A**) Shows the normalized distributions of the trained ANN’s responses for different model classes for all studies used as a priori knowledge for threshold optimization. (**B–E**) Demonstrate the ROC analysis results for inner and outer loops of the nested cross validation (NCV) along with number of voxel-based samples for (training + validation) and test/holdout cohorts for the NMS-ANNs ANNs (Model-1 vs. Models 2&3, and Model-2 vs. Model-3) with 10 different folds. Subfigure 2F shows the inner and outer loops for a tenfold NCV process.

The generalization error and performance of the classifiers were estimated using a tenfold NCV technique^53^. As shown in the subfigure F (Fig. 2), the full dataset was split into 10 non-overlapping folds and two independent loops (outer and inner loops) were defined. In the outer loop, for each trial, the data was split into two folds (training + validation fold and test fold), and for the inner loop, only the validation + training fold was used to select and tune the ANN’s parameters via a conventional k-fold cross-validation technique with Random Permutation Sampling (RPS^54^). For each iteration, the classifier was trained and evaluated^54,55^.

This process was repeated 10 times (Fig. 2F, outer loop). At each repetition, an independent test set was withheld for the estimation of the generalization error and performance of the tuned model (in the inner loop). A Pearson correlation coefficient and an adjusted R-squared (Adj-R^2^) were used as evaluation metrics to quantify the performance of the ANNs for estimation of the estimable PK parameters according to the NMS results. A non-parametric Receiver-Operating-Characteristic (ROC) method^56,57^ was also implemented and the average values of the Area-Under ROC (AUC) was computed to estimate the generalization error and performance of the ANNs for prediction of different nested Model classes. To find the optimal architecture of the ANNs (number of neurons in their only hidden layer), we used the Correct Classification Fraction (CCF, TP + TN)^46,58–60^ for different classes, averaged over all NCV’s k-folds. The average CCF versus different epochs (epoch refers to each trial and error for decreasing the MSE of the ANNs in the batch processing mode) were plotted and the epoch corresponding to 10% of its plateau was used as the stopping epoch or termination error of that subset. The MSE of the ANNs for all *K* subsets at different stopping epochs was calculated and its average value was taken as the ‘stopping error’ for the optimal ANNs^46,58–60^.

### IACUC

This study was approved at the Institutional Animal Care and Use Committee (IACUC) board of Henry Ford Health System and conducted with an approved IACUC # 1509. The animal study of this work was performed and reported in compliance with the ARRIVE guidelines. Also, all methods were performed in accordance with the relevant guidelines and regulations.

## Results

Figure 1 shows the three different physiologically nested conditions and their respective estimable vascular parameters (previously described in the *Calculation of CA Concentration (∆R*_*1*_*) from the Dual Gradient Echo (DGE) Pulse Sequence)*. Subfigure 2A (Fig. 2) shows the normalized distributions of the ANN’s responses for different model classes for all studies used as a priori knowledge for threshold optimization. Subfigures 2B–E (Fig. 2) demonstrate the results for the ROC analyses in inner and outer loops of the nested cross validation (NCV), along with the number of voxel-based samples for (training + validation) and test/holdout cohorts for the NMS-ANNs (Model-1 vs. Models 2&3, and Model-2 vs. Model-3) with 10 different folds. Subfigure 2F (Fig. 2) shows the inner and outer loops for a tenfold NCV process. See the Discussion for inferences drawn from these analyses.

**Figure 3:**
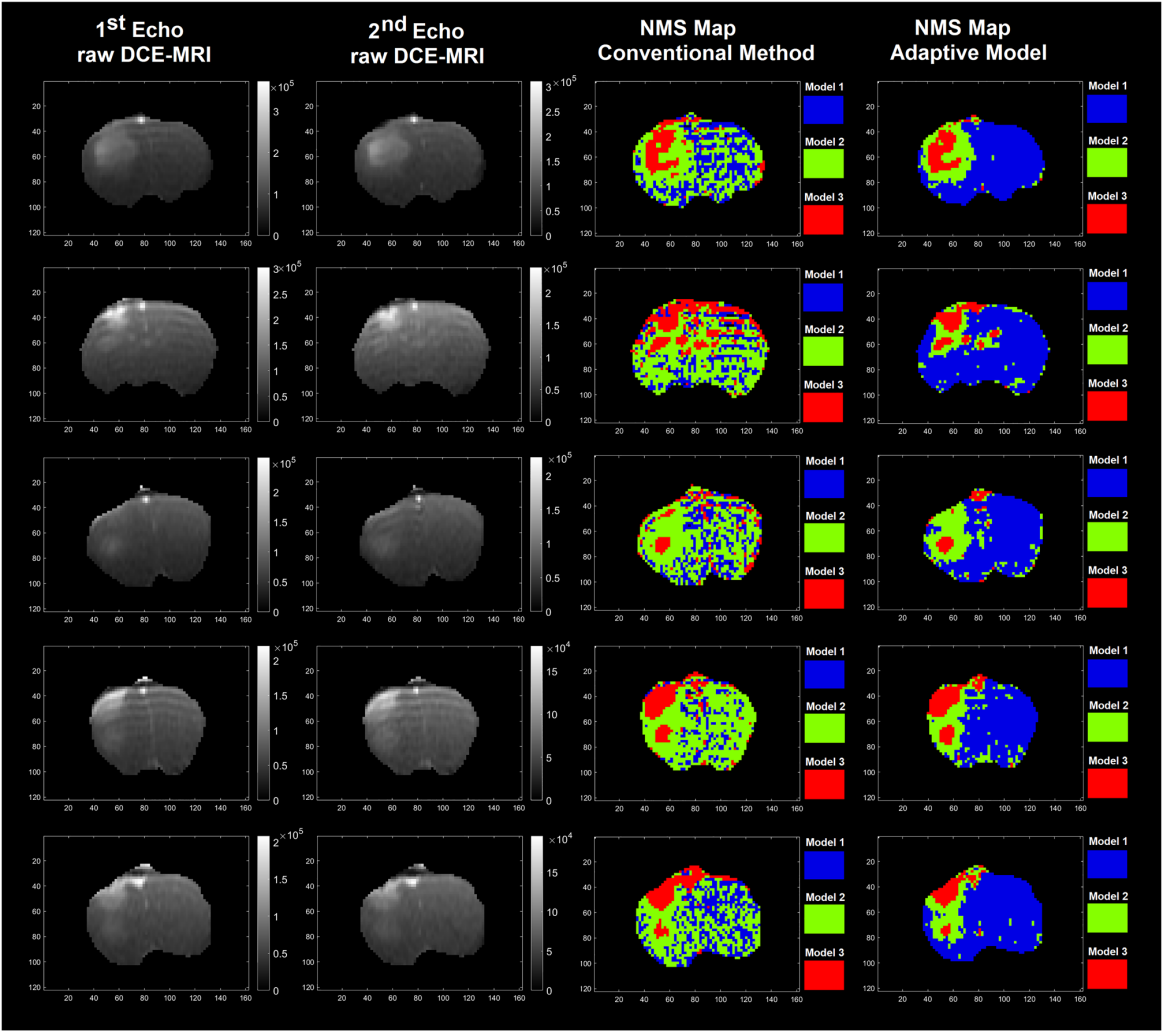
This figure demonstrates 1st and 2nd dual gradient echo images and their corresponding maps estimated by the conventional NMS technique (PK-NMS) and the trained ANNs (ANNs-NMS) for a slice of rat brain for five different animals.

Figure 3 demonstrates 1st and 2nd dual gradient echo images and their corresponding model choice maps estimated by the conventional NMS technique (PK-NMS) and the trained-ANNs (ANNs-NMS) for a slice of rat brain for five different animals.

**Figure 4:**
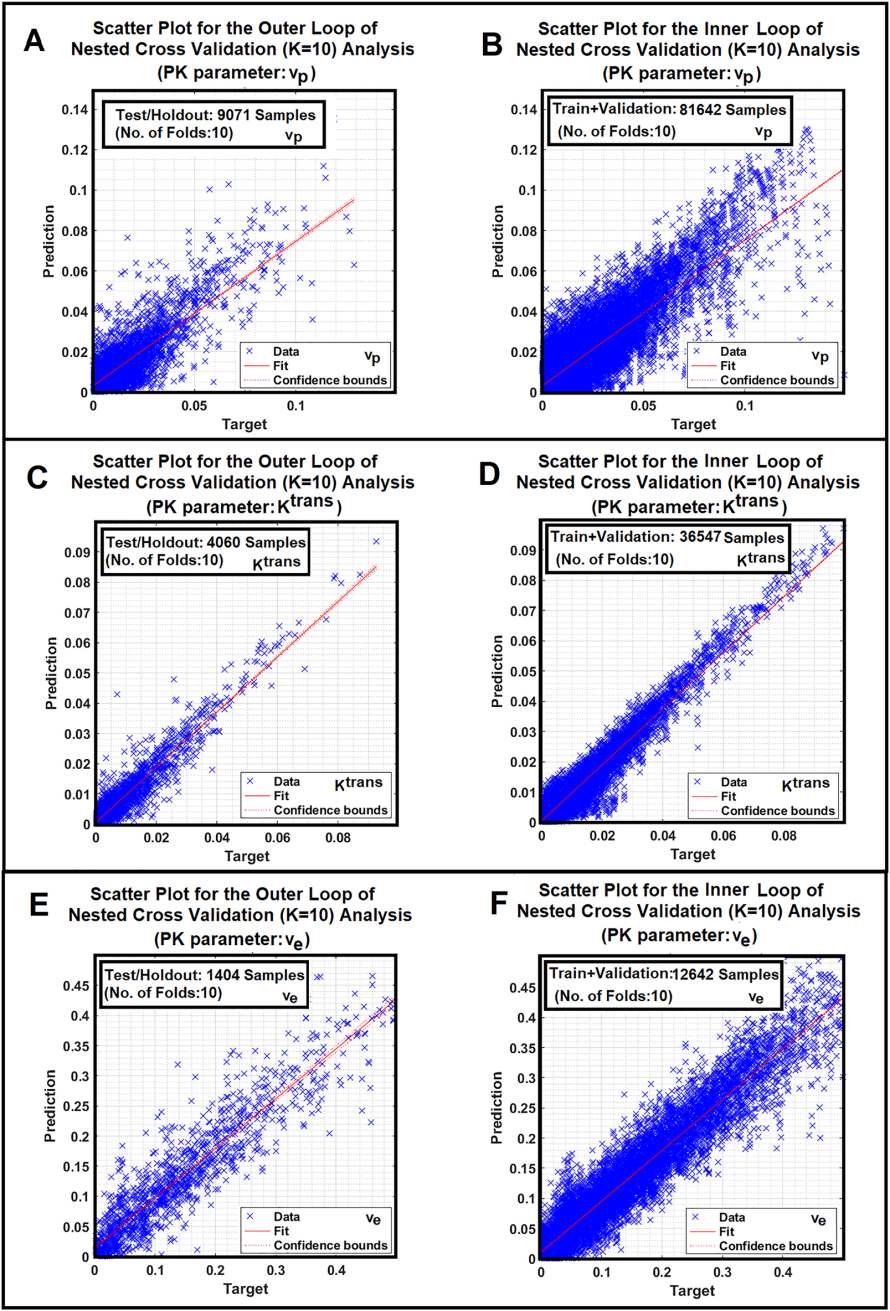
(**A–F**) Demonstrate three pairs (**A,B**)**, **(**C,D**) and (**E,F**) of scatter plots corresponding to the NCV’s inner and outer loops (fold # 10) for training and validation of the three ANNs (ANN-PK) for estimation of three PK parameters (plasma volume, forward transfer constant, and extravascular-extracellular space).

Figure 4 demonstrates three pairs (subfigures 4A,B, 4C,D, and 4E,F) of scatter plots corresponding to the NCV’s outer and inner loops (fold # 10) for training and validation of the three ANNs (ANN-PK) for estimation of three PK parameters (plasma volume, forward transfer constant, and extravascular-extracellular space).

**Figure 5:**
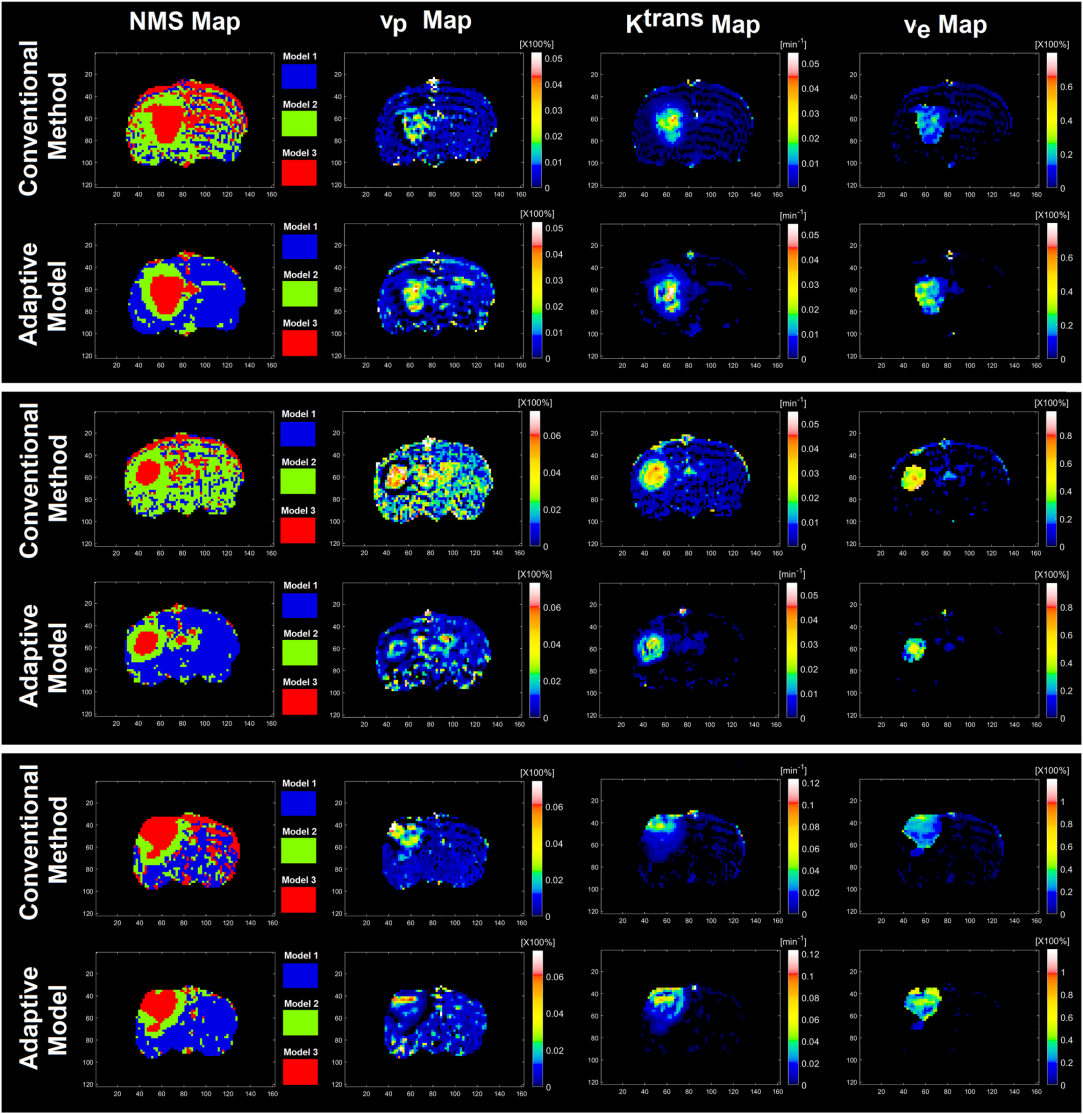
Columns left to right demonstrate NMS maps along with their respective three estimable PK parameters (v_p_, K^trans^, and v_e_) for a slice of rat brain in three different animals with U-251 tumor model estimated by the conventional PK analysis (first, third, and fifth rows) and the trained-ANNs (second, fourth, and sixth rows), respectively. All parametric maps in conventional PK analysis have been masked according to the selected model choice maps created with the F-statistic threshold chosen at the 95% confidence level.

Figure 5 demonstrates (from left to right) NMS maps along their respective three estimable PK parameters (v_p_, K^trans^, and v_e_) for a slice of rat brain in three different animals with U-251 tumor model estimated by the conventional PK analysis and the trained AMs, respectively. All parametric maps in conventional PK analysis have been masked according to the selected model choice maps created with the F-statistic threshold chosen at the 95% confidence level.

Table 1 shows the nested cross validation analysis results for inner and outer loops for all the AMs for prediction of nested model regions (Models 1, 2, and 3) and the estimation of PK parameters. As shown in this table, the performance (Correlation coefficient and Adjusted R-squared for NCV test cohorts) of the AMs for test cohorts were: 0.914 (CI 0.903, 0.925, p < 0.0001)/0.834 (CI 0.815, 0.853), 0.825 (CI 0.814, 0.835, p < 0.0001)/0.720(CI 0.703, 0.737), 0.938 (CI 0.930, 0.946, p < 0.0001)/0.880 (CI 0.864, 0.895), and 0.890 (CI 0.873, 0.906, p < 0.0001)/0.792 (CI 0.762, 0.821) for predictions of nested model regions, v_p_, K^trans^, and v_e_, respectively. The Optimal number of neurons in the only hidden layer of the ANNs were 10, and 7 for the ANNs-NMS and ANN-PK, respectively.

**Table 1:** This table shows the nested cross validation (NCV) analysis results for inner and outer loops for the four adaptive models (AMs for the NMS and for the estimation of PK parameters).

	Pearson correlation coeff. (R)	Confidence interval		p-value	Adj. R-squared	Confidence interval
Inner-loop nested cross validation (NCV)						
Overal performance of nested model selection	0.914	[0.902, 0.925]		p < 0.0001	0.835	[0.814, 0.855]
v_p_, plasma volume fraction	0.864	[0.860, 0.868]		p < 0.0001	0.786	[0.779, 0.793]
K^trans^	0.962	[0.961, 0.964]		p < 0.0001	0.926	[0.923, 0.929]
v_e_, extravascular extracellular volume fraction	0.921	[0.904, 0.938]		p < 0.0001	0.849	[0.817, 0.881]
Outer-loop nested cross validation (NCV)						
Overal performance of nested model selection	0.914	[0.903, 0.925]		p < 0.0001	0.834	[0.815, 0.853]
v_p_, plasma volume fraction	0.825	[0.814, 0.835]		p < 0.0001	0.720	[0.703, 0.737]
K^trans^	0.938	[0.930, 0.946]		p < 0.0001	0.880	[0.864, 0.895]
v_e_, extravascular extracellular volume fraction	0.890	[0.873, 0.906]		p < 0.0001	0.792	[0.762, 0.821]

## Discussion

This study demonstrates the use of knowledge-based AMs for near real-time PK analyses of DCE-MRI data. These AMs characterize the vascular physiology of cerebral tumors directly from raw DCE-MRI data without the need for AIF identification and computationally intensive model-based processing.

The ROC family curves (subfigures 2B–E of Fig. 2) corresponding to 10 different non-overlapping folds lie close to each other in the inner and outer loops of the NCV analyses. This supports the strong association of the extracted features (µ) with model choice, as well as the high classification accuracy of the ANNs. Subfigure 2A (Fig. 2) demonstrates different model overlaps and their skewness in the prediction space. This information is vital for designing a knowledge-based adaptive classifier. A set of optimal classification thresholds can be chosen according to the desired level of misclassification error and error tolerance among different classes/models. The high overlap between Model 1 and 2 (see Fig. 2, subfigure 2A) implies that the misclassification between Model 1 and Model 2 regions is high, presumably due to AIF dispersion and/or delay^61,62^.

This is evident in Fig. 3, where the Model 3 regions estimated by the standard PK-NMS and ANNs-NMS are strongly in agreement, whereas the ANNs-NMS produces Model-1 regions (non-leaky/normal tissues) with less AIF-dispersion effects (less miss-classifications between Model-1 and 2). This figure supports the notion that compared to the conventional PK analysis, trained knowledge-based ANNs can produce more stable maps of vascular parameters and predicted Model-1 regions less impacted by the AIF-dispersion (less miss-classification for Model-1 and 2).

Figure 2 (Subfigure 2A) demonstrates the normalized distributions of the ANN-NMS’s responses for different model classes for all DCE-MRI studies used as a priori knowledge for threshold optimization. As shown in this subfigure, the distributions of the ANN-NMS’s responses for Models 1 and 3 show less overlap compared to Models 3 and 2. Models 1 and 2 show the highest overlap. The overlaps in the response distributions have a number of potential sources, including random-like errors and tapering effects. These latter are not included in the set of models defined as alternatives for fitting the data but appear in the data as minor variations that cannot be included without overfitting. In this study, we assumed that the three models were nested, and that each voxel belonged to a particular model. This allowed us to choose a series of optimal working points (subfigures 2A, output thresholds of 1.73, 1.92, and 2.44, for Models 1 and 2, 1 and 3, and 2 and 3, respectively) for the ANN-NMS to minimize the overlaps among the three-classes and to fine-tune the responses of the ANN-NMS accordingly. This improves the prediction power of the ANN-NMS and directly affects the ANN-PK’s responses by reducing the model mis-classification error at the NMS stage prior to using the ANN-PKs.

### Limitation and future directions

The performance of the ANNs and their predictive power is subject to the values selected for output thresholds. As the training sample size increases, the distribution responses of the ANNs become more stable, resulting in a more accurate selection of these thresholds. As more data becomes available, the adaptive models’ predictive powers and the stability of the nested models predicted by the AMs requires further investigation.

The choice of which models to nest is important. For instance, in the Tofts (two-parameter) versus the three-parameter extended Tofts model (ETM), when the two analyses are applied to the same data there is a typical discrepancy in estimates K^trans^ by as much as a factor of 10. In contrast, when the two-parameter Patlak model is employed as a reduced model we have demonstrated that maps of K^trans^ and v_p_ grade smoothly from the three parameter ETM to the two-parameter Patlak model, to the 1-parameter map of plasma volume. The adaptive model developed in this study was trained on the maps of model selection and associated parameters that were generated using the NMS approach. As in the maps the AMs were trained on, there were no perceptible discontinuities in the final maps of the estimated PK parameters. The PK parameters of different models merged to generate the PK maps and no measurement was assigned/reported for the voxels with non-estimable parameters (such as K^trans^ of Model 1, v_e_ or K_ep_ for Model 1 and 2).

The performance of these AMs can be affected by the amount of the feature regularization on the feature space. A larger sample of data, and features with more information content (less down-sampling of data points) might help in fine-tuning the regularization effect. The extent of regularization and the effective degree of freedom^63^ can be a function of the selected features, sampling rate of the features from the space, inclusion/exclusion of the CA concentration peak in the selected feature, etcetera. Finding an optimal amount of regularization and the effective degree of freedom for construction of these adaptive models requires further investigation.

There is a potential concern for the translation of this study to human and clinical application. The goal of this study was not to develop a model for immediate application to human studies, but to identify signal characteristics that are important in determining PK parameters. The features of the U-251N animal model tumors correlate with human GBM, including a necrotic center, poorly demarcated, infiltrative tumor borders, and an enhanced rim on post-contrast T1-weighted images, which is often observed in human GBMs^64^. Our group has previously shown that in murine models, DCE-MRI measures of vascular permeability^2,3,12,14,16–18,45,47,48,50,51,59,65–71^ agree with those of human studies^14^. Thus, given that cerebral physiology and mechanical properties are relatively constant across species^64,72,73^, the analysis presented in this study can shed light on DCE-MR based components and their values for the characterization of microenvironmental information in tumors.

As noted, the main thrust of this study was to reveal the predictive power and potential of different DCE-MRI based components (1st and 2nd echoes, associated with T_1_ and T_2_* based information, respectively) to estimate PK parameters and to explain the pathophysiological and microvascular properties of normal and cancerous tissues. However, the experimental practices of this paper are not common. Our group is one of the few studying animal tumors using the dual gradient-echo (DGE) pulse sequence. In the construction of vascular permeability and parametric maps, such a sequence allows the separation of T_1_ and T_2_* effects. In an information-centered approach, the data contains additional information concerning the extravascular regime of the tumor which can be explained and modeled by these two components. Unfortunately, we know of no application of the DGE sequence in human DCE-MRI studies.

In this study, down-sampling was done to reduce the computational load and of the feature space complexity (the curse of dimensionality). As a proof-of-concept, we believe that this study supplies support for an adaptive modeling approach to model selection and parametric estimates in DCE-MRI. However, an examination of optimal sampling strategy should be conducted in further studies. The effect of different down-sampling rates on the information content of the extracted features as well as the performance of the constructed AMs is a matter of current study.

Additionally, the sampling rate of the experiment is considered as one of the key elements that would affect the AIF dispersion in non-leaky or normal tissue. For instance, in dynamic susceptibility contrast (DSC) perfusion MRI, sampling rates are in the order of seconds, often with the intent of estimating vascular volumes or flows. The Central Volume Principle tells us that mean transit times for contrast agent in intact brain vasculature are less than 4 s, so a detailed description of the temporal variation of the AIF is important if a deconvolution is to yield reliable estimates of vascular parameters. In DCE-MRI, in the Model 1 tissue, this is also true. An examination of Fig. 6 in Ewing and Bagher-Ebadian^12^ demonstrates this point. On the other hand, mean transit times for contrast agent in the tumor itself (Model 3 region) are one or more minutes; the descriptive equations of leakage are essentially those of a low-pass filter. Thus, the temporal characteristics of the AIF become far less important, while the critical matter of scaling (how much contrast agent enters the tissue) dominates. Generally, the parametric estimates in these studies are in agreement with previous studies performed at a slower sampling rate of 4 s. The increase sampling rate of these studies was adapted with the thought that estimates of blood flow might also be made available. This is a matter of ongoing study in our laboratory.

In recent years, studies have investigated the development of various deep learning and Convolutional Neural Networks (CNNs) to generate more accurate and stable estimates of PK vascular parameters by extracting time-dependent features from DCE-MRI^24,25,28,74–78^. Given DCE-MRI data, accurate estimation of PK parameters strongly relies on appropriate selection of the best PK model to fit the data. In general, for DCE-MRI data analysis, one of the main challenges is to choose the best PK model among competing models to describe the behavior of the time trace of CA concentration in DCE MR experiments. Compared to recent studies^24,25,28,74–78^, one of the novel components of this study is the incorporation of the nested model selection concept into adaptive models for predicting PK parameters.

The fundamental question in MRI procedures that aim to estimate cerebrovascular parameters is the relationship between MR contrast and CA concentration (notated as [CA]). Two common approaches have been to assume that R_2_* is proportional to [CA] (e.g., see Cao et al.^79^), or that R_1_ is proportional. As to the first assumption, in vasculature that leaks, it is clear from a vast literature that competing R_1_ and R_2_* effects strongly affect the relationship between [CA] and the R_2_* MR contrast. Furthermore, R_2_* relaxivity changes drastically as the CA leaves the vasculature and occupies the interstitium^80^. As for R_1_, it has been suggested that restricted water exchange between tissue compartments leads to a biased underestimation of high concentrations^81–85^. Investigations on our part^86,87^, by Buckley et al*.*^88^, and by Springer’s group^13,81^ have shown a strong linearity between [CA] and ΔR_1_, with the effect of restricted water exchange leading only to an underestimation of plasma volume, v_p_. This points to the fundamental utility of MRI measures of ΔR_1_ in estimates of vascular physiology and justifies the use of a shallow ANN for extracting the information content of the time trace of CA concentration from raw DCE-MR data as well as the recruitment of the prior knowledge used in this study. However, it leaves open the question of whether, and under what conditions, Eq. (1) is an appropriate model in various pathologies that can be addressed by the concept of the NMS used in this study.

Unlike similar works^24,28,74–78^ that have used deep learning and CNNs for adaptive modeling that require a large number of samples for their hyper-parameter tuning, training, and validation, the proposed work uses shallow ANNs (with only one hidden layer) with a small number of parameters (compatible with the available sample size: 66 DCE-MRI studies). CNNs and deep learning architectures are capable of learning features as well as the data representations directly from the feature space (input data) with little to no prior knowledge. That's the promise of “no more feature engineering”, which contrasts with other machine learning approaches such as shallow ANNs. Thus, compared to deep learning and CNNs, shallow ANNs (with one hidden layer) might not be the best option for learning feature information that has a high degree of non-linearity. In fact, there is always a tradeoff between a priori knowledge, complexity or information content of the features, amount of training data, the capacity of generalization of the learned models, and the difficulty of training the models. Predictors with smaller numbers of parameters (e.g., shallow ANNs) will have less chance of over-fitting the data but may not learn the details and non-linear information of the features when they encounter complex features. Thus, successful “feature engineering” that can enhance the information content of the features, reduce their correlations, and suppress their unwanted variabilities can play a key role in selecting an appropriate predictive model (*e.g.,* shallow ANNs vs. deep learners). This study strongly supports the recruitment of a shallow ANNs versus deep learners since the ANNs’ generalization errors are low, the ROC family curves and the scatter plots of outer loops in NCV are tight, and the number of neurons in the only hidden layer of the ANNs (ANNs parameters) is compatible with the sample size (66 DCE-MRI studies, 190 features in each feature vector).

The AMs that have been constructed can serve as flexible predictors in that they can be re-trained and fine-tuned based on different optimization methods such as standard NLS fitting, regularized Bayesian estimation methods^89^, etc. Due to their strong generalizability, they can provide high tolerance and robustness against low signal-to-noise, contrast-to-noise ratios, and potential outliers. The optimal threshold tuning based on prior knowledge can help in controlling the amount of regularization for the nested model selection process and reduce the impact of potential heavy tails of the F-Statistic’s distributions on the NMS outcome.

This study demonstrates the use of knowledge-based adaptive models for approximately a real-time PK analysis of DCE-MRI data that characterizes the vascular physiology of embedded tumors directly from DCE-MRI raw data based on a series of physiologically nested models without the need for AIF identification as well as a series of computationally intensive processing. The proposed method can directly estimate the corresponding physiological permeability parameters associated with different physiologically nested models from time trace of raw DCE-MR signal intensity, which eliminates several intermediate computation steps of the conventional pipeline.

Model simulation to generate synthetic data was not performed in this study. Instead, we connected the raw-DCE-MRI information of tumor and its surrounding normal tissues with the conventional NMS-PK analysis results. This allows the incorporation of many tapering effects, i.e., systematic, and random-like errors, in our adaptive modeling. These tapering effects would not usually be included in model simulations, are not easily evaluated in those processes, and thus might cause a significant bias in evaluating the performance of an AM in its application to data sampled in real-life procedures.

We assumed a priori data could be described voxel-by-voxel by one and only one of three nested models. This allowed us to choose a series of optimal working points for the adaptive models to suppress the impact of tapering effects on their PK parameter estimations. In Fig. 5 (subfigures A and B), the three PK maps estimated by the ANNs appear more heterogeneous compared to their corresponding maps from the conventional PK analysis. Similarly, the NCV-based evaluation results also show the differences between the estimations of the two methods (conventional PK analysis versus ANNs shown in Table 1).

A key question that arises from the heterogeneity of the ANN’s parametric maps is why the conventional PK analysis results slightly differ from the ANNs’ estimations since the conventional analyses are used as the source of truth for the training of the ANNs. One reason lies in the differences in the model choice maps generated by the ANNs-NMS versus the conventional NMS technique, albeit there appears to be good agreement in the tumor itself. Figures 4 and 5 demonstrate that the prediction of the model choice map produces a far less noisy map than the conventional analysis, particularly in model 1 and 2 regions. This result is consistent with a priori knowledge that contrast agent doesn’t leak out of the microvasculature in normal brain tissue.

In the Model 3 regions, however, the ANN parametric estimates show greater spatial heterogeneity. This may be due to regularization effects for the three independent adaptive models (ANNs-PK). In this study, construction of three independent and decoupled adaptive models for estimation of different PK parameters increased the chance of the regularization effect on each of the PK parameters during the ANNs-PK training. To keep this regularization effect consistent among the three adaptive models (PK predictors), the same effective degree of freedom and complexity level were used for these adaptive models (ANNs-PK). Another source of heterogeneity in individual voxels may lie in the choice of not including an AIF as an overall input function for all voxels.

To minimize the effects of potential feature non-uniformities from different animal sub cohorts/folds, we used a higher number of folds (K = 10) in the NCV process compared to the default number (K = 5) recommended by the literature^90–92^. Model development and validation without nested CV uses the same data to train and tune the adaptive model parameters and evaluate model performance. Information may thus “leak” into the model and overfit the data. The magnitude of this effect is primarily dependent on the size of the dataset and the stability of the model. To minimize this problem, we implemented a nested CV technique, in which the testing dataset is held-out during training and validation at each of multiple folds. The PK parameter estimates obtained by the proposed approach yields improved statistically significant differences between different tissue types, which can ultimately allow better classification and a more conservative generalization error estimation for discrimination of model choice regions in PK analysis.

One great advantage of the approach to parametric estimates derived from DCE-MRI data is the short time to a usable answer. Compared to conventional fitting methods, the PK parameter inference including model selection with the trained ANNs is computationally faster and takes a fraction of a second (Core (TM)-6700HQ, CPU @2.60 Hz) on an entire 3D DCE-MRI volume (for entire animal brain with 3 full slices). This suggests that these procedures can be adapted to human studies with larger data sets and still incorporated into a clinical pipeline for rapid presentation for evaluation and treatment planning. Thus, further investigation of the effects of different ANN’s architecture, training the models with larger sample size and training algorithms as well as their impact on the estimation of the PK parameters is warranted.

## Conclusion

This study demonstrates an application of knowledge-based Adaptive Models to improve robustness and computation speed, and to reduce the biases introduced by both random-like errors and tapering effects (particularly due to errors in estimating the AIF) that appear in conventional approaches to estimating DCE-MRI vascular parameters. Nested Model Selection was retained as a novel and essential element in the analysis approach, leading to stable estimates of vascular parameters. Importantly, AMs produced estimates of vascular parameters without the necessity of AIF identification. Because of its rapid estimation of vascular PK parameters directly from the DCE MR information without complex mathematical modeling, the AM approach can serve as an efficient tool to assess tumor vascular permeability to facilitate small animal brain tumor research studies. We believe that the AMs introduced in this pilot study, if fine-tuned and translated to human use, can significantly improve the clinical diagnostic and prognostic decisions made for patients with GBM.

## Data Availability

All imaging data used in this investigation along with programming codes and results are available and can be shared upon request to the corresponding and senior authors.
